# Mendelian randomization applied to pharmaceutical use: the case of metformin and lung cancer

**DOI:** 10.1093/ije/dyaa059

**Published:** 2020-05-01

**Authors:** James Yarmolinsky, Caroline J Bull, Venexia M Walker, Aayah Nounu, George Davey Smith

**Affiliations:** MRC Integrative Epidemiology Unit, Population Health Sciences, Bristol Medical School, University of Bristol, Bristol, UK

Zhou *et al*. recently reported findings from a Mendelian randomization analysis aiming to examine the causal relationship between metformin use and lung cancer risk.^1^ This is a topical question because of previously reported associations between use of metformin, a commonly prescribed drug for the treatment of type 2 diabetes, and lower cancer risk across several anatomical sites, including lung cancer.^2^^,^^3^

In lieu of an established drug target of metformin, Zhou *et al*. used genetically-proxied measures of growth differentiation factor 15 (GDF15), a cytokine previously reported to be strongly associated with metformin use,^4^ to ‘assess the causal relationship between metformin use and lung cancer occurrence’. The authors interpreted their findings as indicating no evidence of a causal relationship between these traits. We have some methodological and interpretative concerns regarding the analyses presented in this paper.

In order to examine the causal effect of metformin on lung cancer risk using GDF15 as a marker of metformin use, it is necessary to assume that (i) metformin use affects GDF15 levels and (ii) any effect of metformin on lung cancer is entirely mediated through GDF15 levels. Although we believe the first assumption to have reasonable face validity, we believe that the second assumption is likely to be violated.

The identification of GDF15 as a potential biomarker of metformin use was first reported in a cross-sectional analysis of metformin use and 237 serum biomarkers using baseline data from 8401 participants enrolled in the Outcome Reduction with Initial Glargine Intervention (ORIGIN) trial.^4^ The large effect size for this association observed in models adjusted for clinical factors and other serum biomarkers (Odds ratio of metformin use: 3.94; 95% confidence interval: 3.59–4.33 per standard deviation increase in GDF15) provides some assurance that this finding is unlikely to be largely driven through residual confounding (e.g. due to unmeasured or imprecisely measured confounders). Though GDF15 appears to be a plausible mediator of some of the antidiabetic effect of metformin (e.g. by reducing body weight), reverse causation (i.e. higher pre-baseline GDF15 levels increasing the likelihood of subsequent metformin prescription) cannot be ruled out given emerging evidence to suggest an effect of nutritional imbalance on circulating GDF15 levels.^5^^,^^6^ At a minimum, it would have aided the reader in interpreting conclusions from their analysis if Zhou *et al*. had more clearly conveyed the novelty of the findings reported in the ORIGIN trial and, thus, the provisional nature of the hypothesized relationship between metformin use and GDF15.

Even if the assumption that metformin use affects GDF15 were to hold, we believe that it is incorrect to interpret a Mendelian randomization analysis of GDF15 on lung cancer risk as being equivalent to assessing ‘causality between metformin use and lung cancer incidence’, as claimed by Zhou *et al*. This is because GDF15 levels represent only one of several hypothesized mechanistic pathways through which metformin use may influence subsequent lung cancer risk.^7^ For example, metformin has been shown to influence both (i) tumour metabolism through inhibition of mitochondrial complex I and (ii) tumour development through altered systemic metabolism (e.g. through lowering blood glucose via reduced hepatic gluconeogenesis, in turn reducing plasma insulin levels).^8^^,^^9^ Both mechanisms involve activation of AMP-activated protein kinase (AMPK) by metformin, an inhibitor of the mammalian target of the rapamycin (mTOR) pathway, which reduces cell proliferation and induces cell cycle arrest and apoptosis.^7^ In addition, it is possible that GDF15 may simply act as a biomarker of metformin use and may not provide any information on the mechanism of action of metformin or its health effects. We therefore believe that the Mendelian randomization analysis presented by Zhou *et al*. would more accurately be described as an analysis of the effect of GDF15, rather than metformin use, on lung cancer risk.

Finally, Zhou *et al*. constructed an instrument to proxy GDF15 from five genetic variants all of which were in mild to high linkage disequilibrium (*r*^2^ = 0.25–0.80) with at least one other variant in the instrument. The two sensitivity analyses employed to test for evidence of violations of Mendelian randomization assumptions either cannot be performed using correlated variants (weighted median estimation) or are susceptible to violation of the InSIDE (Instrument Strength Independent of Direct Effect) assumption (MR-Egger) even if correlations were considered in the model for this latter analysis. Effect estimates generated from the weighted median estimate could be biased either toward or away from the null, depending on the genetic correlation structure between individual variants. Consequently, the robustness of the overall null finding reported by the authors to bias from negative horizontal pleiotropy (attenuation of the effect estimate toward the null due to the presence of genetic variants influencing lung cancer through pathways independent to GDF15) is unclear.

Mendelian randomization remains a potentially powerful approach for the discovery, development and repurposing of pharmacological agents for disease prevention and treatment.^10^ However, careful attention must be paid to both the interpretation and design of such studies in order to avoid drawing erroneous conclusions.

## Funding

J.Y., C.J.B. and A.N. are supported by a Cancer Research UK Programme Grant [The Integrative Cancer Epidemiology Programme] (C18281/A19169). All authors are members of the MRC Integrative Epidemiology Unit which is supported by the Medical Research Council and the University of Bristol (MC_UU_12013/1–9) (https://mrc.ukri.org/, http://www.bristol.ac.uk/).

### Conflict of interest

None declared.
